# Core components and strategies for suicide and risk management protocols in mental health research: a scoping review

**DOI:** 10.1186/s12888-020-03005-0

**Published:** 2021-01-07

**Authors:** Katye Stevens, Vivetha Thambinathan, Elisa Hollenberg, Fiona Inglis, Andrew Johnson, Andrea Levinson, Soha Salman, Leah Cardinale, Brian Lo, Jenny Shi, David Wiljer, Daphne J. Korczak, Kristin Cleverley

**Affiliations:** 1 The Margaret and Wallace McCain Centre for Child, Youth and Family Mental Health, Centre for Addiction and Mental Health, Toronto, Ontario Canada; 2 The Western Centre for Public Health and Family Medicine, Western University, London, Ontario Canada; 3 Office of Education, Centre for Addiction and Mental Health, Toronto, Ontario Canada; 4 Library Services, Centre for Addiction and Mental Health, Toronto, Ontario Canada; 5 Humber Libraries, Humber College, Toronto, Ontario Canada; 6 Department of Psychiatry, Faculty of Medicine, University of Toronto, Toronto, Ontario Canada; 7 Department of Psychiatry, Centre for Addiction and Mental Health, Toronto, Ontario Canada; 8 Department of Psychiatry, Hospital for Sick Children, Toronto, Ontario Canada; 9 Lawrence S. Bloomberg Faculty of Nursing and Department of Psychiatry, University of Toronto, Toronto, Ontario Canada; 10 Institute of Health Policy, Management and Evaluation, University of Toronto, Toronto, Ontario Canada; 11 Information Management Group, Centre for Addiction and Mental Health, Toronto, Ontario Canada; 12 Centre for Excellence in Economic Analysis Research, St. Michael’s Hospital, Toronto, Ontario Canada; 13 Education, Technology and Innovation, UHN Digital, University Health Network, Toronto, Ontario Canada

**Keywords:** Suicide and risk management protocol, Mental health research, Scoping review

## Abstract

**Background:**

Suicide and risk management protocols in mental health research aim to ensure patient safety, provide vital information on how to assess suicidal ideation, manage risk, and respond to unexpected and expected situations. However, there is a lack of literature that identifies specific components and strategies to include in suicide and risk management protocols (SRMPs) for mental health research. The goal of this scoping review was to review academic and grey literature to determine core components and associated strategies, which can be used to inform SRMPs in mental health research.

**Methods and analysis:**

The methodological framework outlined by Arksey and O’Malley was used for this scoping review. The search strategy, conducted by a medical librarian, was multidisciplinary and included seven databases. Two reviewers independently assessed eligibility criteria in each document and used a standardized charting form to extract relevant data. The extracted data were then examined using qualitative content analysis. Specifically, summative content analysis was used to identify the core components and strategies used in SRMPs. The data synthesis process was iterative.

**Results:**

This review included 36 documents, specifically 22 peer-reviewed articles and 14 documents from the grey literature. Five core components of SRMPs emerged from the reviewed literature including: training; educational resources for research staff; educational resources for research participants; risk assessment and management strategies; and clinical and research oversight. Potentials strategies for risk mitigation within each of the core components are outlined.

**Conclusions:**

The five core components and associated strategies for inclusion in SRMPs will assist mental health researchers in conducting research safely and rigorously. Findings can inform the development of SRMPs and how to tailor them across various research contexts.

**Supplementary Information:**

The online version contains supplementary material available at 10.1186/s12888-020-03005-0.

## Background

Suicide and risk management protocols (SRMPs) in mental health research “*document procedures for identification of, and care for, suicidal participants within the context of a research study*” ([1], p.2). Importantly, SRMPs also aid in ensuring patient safety, provide vital information on how to assess suicidal ideation and manage other types of risk, and respond to adverse events [2, 3]. Mental health researchers and their research staff may need to intervene in risk situations such as suicidal ideation, and disclosing of harm to self and/or others during research visits and/or interviews. Without broader guidelines on how to prepare for, and manage risk, within mental health research protocols, both researchers and Research Ethics Boards (REBs) may struggle with creating individualized SRMPs that meet the needs of study participants. While it is necessary to tailor these standardized procedures for suicide and risk management to local clinical and research contexts, study populations and study-specific needs, researchers would benefit from evidence-informed guidelines that specify the core components of a SRMP and guide researchers [1].

Researchers communicate a study’s alignment with national and international safety and ethical standards through the development of SRMPs. These protocols should be consistent with the content of the International Conference on Harmonization who developed the Guidelines for Good Clinical Practice [4]. Widely recognized and used in clinical trials, standardized protocols describe safe practices to: (a) develop consistency within research teams; (b) identify roles and responsibilities during specific events; and (c) promote the smooth functioning of the day-to-day activities of research projects [5]. There is a multiplicity of terms in the literature that refer to SRMPs including standard operating procedures (SOPs), safety protocols, crisis protocols, and safety management procedures, among others. To address this inconsistency in terminology and be inclusive of suicide risk and other types of risk, we will use the term SRMP throughout this review.

It is important to acknowledge that the risk of suicide may require intervention as a part of the SRMP, and participants may require a clinical intervention [2]. This unknown and controversial territory of risk for participants can lead to a complex and ambiguous REB review process that can precipitate confusion between REBs and researchers. To assist research teams in navigating the REB review process, there has been some literature addressing effective ways to communicate potential risks in mental health research using SRMPs [3, 6]. However, according to Schatten et al. [2], there is substantial variability in the identification and management of suicide risk amongst different studies. For individual researchers and their ethics committees, decision-making is typically based on the particular sample and research methodology.

There have been significant collaborative efforts to standardize REB processes across different countries around the world. In the United Kingdom, Research Ethics Services is the governing body that ensures standardization of ethical research practices across all established research committees. This includes establishment of a United Kingdom-wide set of SOPs and providing operational guidelines [7]. In Australia, the ‘national statement’ has been developed by health and research organizations within the country that defines regulations for ethical practices and thus, assures standardization across research bodies [8]. Similar to Australia, organizations such as the Food and Drug Administration in the United States have developed regulations as well as non-binding recommendations regarding ethical practices for institutions and Institutional Research Boards [9]. In Canada, through open access, the Canadian Association of Research Ethics Boards and Network of Networks provide collaboratively developed, standardized, Canadian REB SOPs [10]. These standardized procedures are specific to health sciences REBs and aim to encourage the maintenance of a single universal standard for REBs. Such efforts are in response to reported variations among REBs in assessing the same clinical research protocol [11–13]. In some cases, there were no requirements outlined by a REB for researchers to implement a SRMP. Thus, once a study has received REB approval, it is at the discretion of research teams to develop relevant SRMPs for mitigating risk and prioritizing the safety of research participants. This activity is particularly important where there is a real or perceived risk, including studies where the population may be at higher risk of self-harm or suicidal ideation, or death from suicide [14, 15]. Having standardized expectations for the content of SRMPs across REBs is in the best interest of researchers, REBs, and research participants [16]. Yet, a preliminary review of the literature revealed a lack of studies that identified specific components and strategies to include in SRMPs for mental health research where there is a risk of suicide and harm to self and others.

To address this knowledge gap, a scoping review was conducted on SRMPs, used in mental health research documents that assess and manage risk and safety for research participants. The objective of this review was to identify (a) core components and (b) strategies of SRMPs to assess and manage the risk and safety of participants in mental health research. The findings will be used to investigate, review, and inform the development of recommended guidelines that clinicians, researchers, REBs, and academics can adopt when developing SRMPs for their mental health research studies where there is a risk of harm.

## Methods / analysis

A scoping review was conducted to identify key gaps in the literature and broadly scope existing scientific and grey literature to provide a greater understanding of key constructs in this area of research [17]. The scoping review protocol followed the methodological framework outlined by Arksey and O’Malley [18]. This five-stage approach is described below. To ensure transparency and methodological rigour throughout this scoping review, this protocol followed the Preferred Reporting Items for Systematic Review and Meta-Analyses extension for Scoping Reviews (PRISMA-ScR) checklist [19].

### Stage 1: identifying the Research question

The following research question was the foundation for this scoping review:

1. What are the (a) core components and (b) strategies of SRMPs to assess and manage the risk and safety of adult (> 16 years of age) participants in mental health research?

Fixsen et al. [20] define core components as *“the most essential and indispensable components of an implementation practice or program” (p.24)*. This definition which was utilized by Cleverley et al. [21] in a recent mental health scoping review will also be used to define core components and strategies in this scoping review.

### Stage 2: identifying relevant documents

#### Eligibility criteria

All research studies (experimental, quasi-experimental, observational, qualitative) and grey literature (standard operating procedures, guidelines, narrative reviews, conference papers and proceedings, government reports, community agency/group reports, editorials, policy documents, theses) examining protocols in mental health research for risk and safety assessment and management were included. We did not restrict studies based on publication date or study design. The population was restricted to those aged 16 years and up, to make the important distinction between mental health research studies involving children and adults. Due to time constraints and financial resources, we also restricted the language of studies to English. Eligible studies and documents were specific to mental health and included the broad term ‘standard operating procedure’ or a synonymously related concept (e.g., SRMP, crisis protocol, standard work procedure).

#### Search strategy

**Fig. 1:**
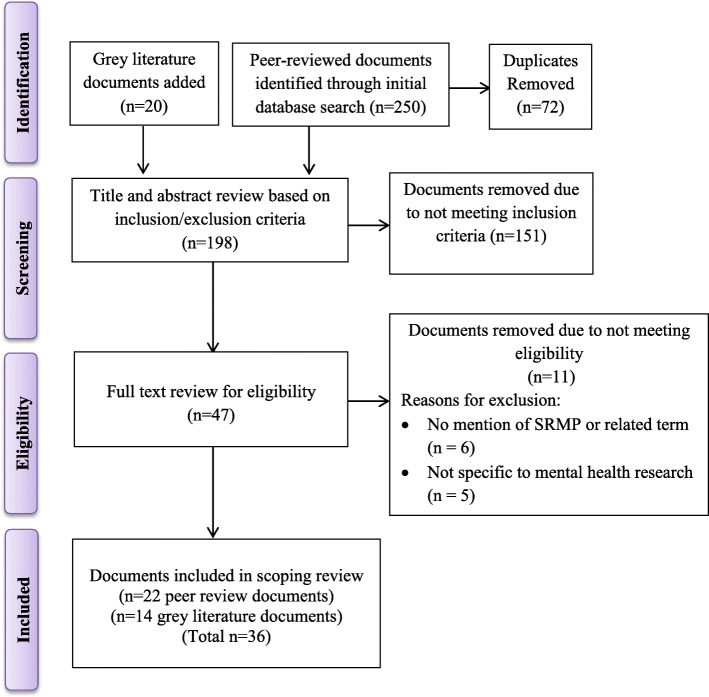
PRISMA diagram

The search strategy was iteratively developed by an experienced medical librarian (FI) who has extensive expertise in mental health literature searches in consultation with the research team. This strategy was initially built in Medline and then translated as required for the other databases (see Additional file 1 for the full Medline strategy). We searched the following databases: Medline (including Epub Ahead of Print, In-Process and Other Non-Indexed Citations), EMBASE, PsycINFO, Cochrane Database of Systematic Reviews (all OVID interface), CINAHL (EBSCO), Sociological Abstracts (ProQuest), and Web of Science Core Collection. This specific range of databases produced a comprehensive and multidisciplinary search. ‘Standardized operating procedures’ and ‘risk’ are defined in different ways within mental health research and across disciplines that are of interest. As a result, the search strategy included both subject headings and keywords related to the concepts of SOPs, research subjects and risk of harm (see Additional file 1). In addition, an extensive grey (unpublished) literature search was conducted (VT) through consultation with an experienced research librarian and informed by “*Searching the Grey Literature: A Handbook for Searching Reports, Working Papers, and Other Unpublished Research*” [22]. This search was conducted using the Open Gray Repository, Google Advanced, ProQuest Dissertations and Theses Global, and the methods outlined in the Canadian Agency for Drugs and Technologies in Health search tool, ‘Grey Matters’ [23]. Experts in the field were also contacted to identify any additional relevant documents. The results from the searches were exported into the citation manager EndNote to retrieve bibliographic details and perform de-duplication. A total of 178 peer reviewed documents and 20 grey literature documents were retrieved from the database search after removing 72 duplicates. (see Fig. 1 for PRISMA diagram).

### Stage 3: study selection

Eligibility criteria were pilot tested on a random sample of 25 abstracts until substantial inter-rater agreement was reached between the two reviewers (VT, KS); κ ≥ 0.70 [24]. The eligibility criteria were continuously refined to ensure both reviewers were aligned. The study selection process underwent two layers of screening: (1) title and abstract and (2) full-text review. In both stages, two reviewers (VT, KS) independently reviewed the articles and determined eligibility using the set inclusion / exclusion criteria. Throughout this process, both reviewers used Covidence [25] - an online software recommended by Cochrane that serves as a screening and data extraction tool for literature reviews. When consensus was not achieved, the two reviewers consulted with the senior author (KC) to resolve disagreements. The search strategy results and eligibility screening process are reported in detail using the PRISMA flow diagram for scoping reviews [19]. Following title and abstract screening of 198 documents, 151 were removed as they did not meet the inclusion criteria. A total of 47 full-text documents were examined; of these, 11 were ineligible (see Fig. 1 for PRISMA diagram) leaving 36 for inclusion in this review.

### Stage 4: data items and data collection process

Based on our research question, a standardized charting form was developed by our research team to capture relevant data in Excel. The following information was extracted from each document and entered into the form:

- Descriptive information about the document: authors, title of publication, year of publication, country of origin, publication type, aims / purpose / goals of study, research study design and methodology, study population and setting, disease type, method of data collection (e.g. in-person, online), and study limitations.
- Suicide and risk management protocols: presence of SRMP, term used in document (e.g. SRMP, SOP), the core components of the SRMP, and the strategies within the SRMP indicating how the SRMP manages risk and safety for research participants and research staff.

Prior to data extraction, the data charting form was piloted with two reviewers (KS, VT) independently charting three articles, followed by meeting with the research team. This process was iterative as the research team revised the form as needed throughout data extraction as new themes emerged and new categories of interest were identified.

### Stage 5: synthesizing and reporting the results

The extracted data from the data charting form underwent qualitative content analysis [26]. Content analysis generally refers to a family of analytic approaches that provide meaning to the content of the data [27]. Summative content analysis, a specific type of content analysis, involves determining the frequency of key words or content that then permits interpretation of the data within a specific context (e.g. core components and strategies use in SRMPs) [27]. This analysis was appropriate given the nature of available data extracted from the scoping review documents. The overall synthesis process was iterative to ensure that we captured data emerging from the academic and grey literature.

## Results

### Characteristics of documents

A total of 36 documents (22 peer reviewed articles and 14 grey literature documents) were included in this scoping review. The 22 peer reviewed articles were published between 1988 and 2020 with 9/22 (41%) being published in the past 5 years. In total, 17/22 (77%) articles were from the United States with the remainder from Canada (2), Australia (1), England (1), and the Netherlands (1). Of the 22 peer reviewed articles, there were qualitative (*n* = 4), quantitative (*n* = 3), and mixed (qualitative and quantitative) (*n* = 7) methods utilized, and one was a meta-analysis. The remaining (*n* = 7) were commentary or recommendation documents. The grey literature consisted of guidance documents (*n* = 9) and reports (*n* = 5). All 14, except one unknown, grey literature documents were published between 2006 and 2019 with 7/14 (50%) being published in the past 5 years. In total, 9/14 (64%) documents were published from the United States with the remainder from Canada (2), Australia (1), and England (1). All 36 documents focused on mental health research, as per the inclusion criteria. Study populations were most commonly adults diagnosed with a variety of mental health issues including depression, suicidal ideation, bipolar disorder, schizophrenic disorder, borderline personality disorder, amongst others. The 36 documents specified either hospital or community health settings. Multiple data collection methods were utilized with the vast majority including face-to-face methods (e.g. self-report paper-based measures or qualitative interviews), telephone communication, and online methods (e.g. use of electronic safety and risk management protocol, online questionnaires).

### SRMP Core components and strategies

In this section, core components and strategies of a SRMP that were identified in the reviewed documents will be described (see Table 1). Consistent with the definition by Fixsen et al. [20], core components in this review were identified as those components that were essential in the development of a protocol to successfully assess and manage suicide and risk for participants in mental health research. A total of five core components were identified, including: (1) training; (2) educational resources for research staff; (3) educational resources for research participants; (4) risk assessment and management strategies; and (5) clinical and research oversight. For each core component, a variety of implementation strategies were identified.

**Table 1:** Table 1

Core components	Strategies	References	
		Peer-reviewed	Grey
(1.0) Training	(1.1) Research staff training	[2, 3, 6, 28–32]	[33–36]
	(1.2) Team building	[29]	
	(1.3) Establishing relevant experience, delineating roles, and ensuring qualifications for the research team	[3, 6, 15, 37, 38]	[39–41]
	(1.4) Follow-up and debriefing with research staff	[2, 6, 16, 28, 30, 37, 38]	[40]
(2.0) Educational Resources: Research Staff	(2.1) Resources, procedures, and guidelines	[29]	[42]
	(2.2) Flow charts and algorithms	[1, 3, 6, 14, 29, 30]	[43–46]
	(2.3) Electronic prompts/Pop-up alerts	[30]	[46]
	(2.4) Templates and general principals	[1, 16, 47]	[33]
(3.0) Educational Resources: Research Participants	(3.1) Written materials	[3, 15, 16, 32, 48–50]	[33, 35, 36, 42, 43, 51]
	(3.2) Immediate support services	[1, 2, 14, 47, 49, 50, 52, 53]	[34, 40, 43, 44, 46]
	(3.3) Available mental health services	[2, 3, 14, 16, 49, 52–54]	[41, 43]
	(3.4) Social support person	[2, 3, 52]	[42]
(4.0) Risk Assessment and Management Strategies	(4.1) Implementation of a structured and explicit suicide and risk management protocol	[6, 14–16, 29, 31, 32, 38, 50, 52, 55]	[34, 36, 51]
	(4.2) Identification of risk level	[3, 30, 37, 47, 50, 54]	[34, 36, 39, 42, 44, 51]
	(4.3) Utilization and scoring of validated risk assessment measures	[14, 30, 32, 37, 50, 52, 55]	[33, 35, 43, 46]
(5.0) Clinical and Research Oversight	(5.1) Use of a data and safety monitoring board or a data monitoring plan	[1–3, 6, 29, 31]	[56]
	(5.2) Safety and risk monitoring	[6, 16, 29, 37]	[46, 56]
	(5.3) Clinical back-up during research assessments	[1, 6, 14, 29, 30, 37, 38, 50, 54]	[44, 56]

### Component 1.0: training

Training included strategies to prepare researchers and research staff with the knowledge, skills, and competency to assess and manage risk of participants in mental health research. Of the 36 documents reviewed, 19 (53%) included training as a core component (see Table 1). A total of four different training strategies were described across the documents. Specific training strategies described in the documents included: (1.1) research staff training; (1.2) team building; (1.3) establishing relevant experience, delineating roles, and ensuring qualifications for the research team; and (1.4) follow-up debriefing with staff. Each of these are described below with reference to key supporting documents.

### Training strategies

**1.1:** ***Research staff training*:** The reviewed documents indicated that research staff need to be trained on suicide and risk assessment [2, 3, 6, 28–35] and in the implementation of a safety protocol [36]. Specifically, there ought to be a plan for initial and ongoing training on the use of SRMPs and how to respond in different situations [6]. Several documents (see Table 1) referred to training methods that focused on both the research participants and the trainer. For example, Campbell et al. [29], conducted an extensive three-day workshop for all staff collecting study data. The workshop included educational and interactive sessions that provided detailed information regarding conducting suicide risk assessments and role-playing activities where trainees participated in mock interviews with research staff. Herbeck Belnap et al. [30] had a study psychologist conduct a one-day staff training session with study staff about suicidal ideation and embedded time to practice using the SRMP [30]. Lastly, Hom et al. [3] included reading key scientific papers, shadowing and observation experiences, reviewing training videos with mock participants, annual evaluation of competence in risk assessment using standardized role play, routine reviews using audiotapes, video tapes or observation by a clinical supervisor.

**1.2:** ***Team building*:** Only one author described the importance of team building as a strategy. Ensuring the research study team is developing cohesively was suggested by Campbell et al. [29] who included strategies such as; rapport-building with research participants and cultivating relationships with participating sites, to facilitate identification and testing of suicide assessment and management strategies.

**1.3:** ***Establishing relevant experience, delineating roles, and ensuring qualifications for the research team*:** Determining the experience, qualifications, and knowledge of the research team is essential [3, 6, 15, 37–41]. Based on variability in the knowledge and experience of members of the research team, individualized training (e.g. implementation of study procedures and protocols, administration of study assessments and interpretation of results) is necessary so that the research team members have the knowledge to deal with suicide risk [15, 38, 41]. To avoid potential confusion regarding the assessment and management of research participants, study protocols should clearly outline roles and boundaries for research staff [3]. Ghahramanlou-Holloway et al. [37] explicitly stated which staff member was to intervene at each point in the assessment. Upon awareness of potential suicide risk for participants, study assessors (who are defined as trained staff in administering measures) were instructed to contact the study principal investigator (PI) immediately. Similarly, Littlewood et al. [38] mentioned their study was conducted by research staff members with prior mental health research experience. For researchers conducting psychiatric research, a formal education in research ethics to address complex issues is necessary [39]. Most importantly, the research team lead (i.e. PI; research manager) must be trained in identifying elevated risk (e.g. through suicide assessments and clinical judgment) [15].

**1.4:** ***Follow-up and debriefing with research staff*:** Follow-up and debriefing with research staff and study participants is critical when training research staff on how to implement a SRMP and after it has been implemented. Staff need an opportunity to troubleshoot, ask questions, and seek ongoing training, advice, and feedback [2, 6, 16, 28, 30, 37, 38, 40]. Examples of follow-up and debriefing procedures consisted of: (a) one-on-one or small group discussion between the PI and research staff [28]; (b) a staff discussion with the study psychologist or other clinician [30]; and (c) researchers (e.g. PI or Co-Investigator) set aside specific times for debriefing and problem solving with research staff on AEs [16].

### Component 2.0: educational resources - Research staff

Educational resources in the reviewed documents targeted two different groups of stakeholders: research staff and research participants. Strategies directed specifically at research staff included: (2.1) resources, procedures, and guidelines; (2.2) flow charts and algorithms; (2.3) electronic prompts/pop-up alerts; and (2.4) templates and general principals. Of the 36 documents reviewed, 14 (39%) included educational resources for research staff (see Table 1). Each strategy is described in more detail below with reference to the key documents describing them. Strategies directed specifically at research participants are discussed in Component 3.0.

### Educational resources - Research staff strategies

**2.1:** ***Resources, procedures, and guidelines*:** Research staff strategies indicated in this review included educational resources, procedures and guidelines [29, 42]. Campbell et al. [29] suggested the use of an “On Call Clinician Resource Guide” which consisted of local resources (e.g. local research site clinical resources) and safety management procedures (e.g. risk management policies and procedures for documentation and communication) for on call study clinicians to use when needed. This “On Call Clinician Resource Guide” also consisted of contact information for on call personnel in the form of a wallet card for easy access [29]. Luxton et al. [42] recommended the use of tele-practice guidelines to determine appropriateness of patients receiving care as well as SOPs consisting of a series of steps to assess suicide risk level and respond to psychiatric emergencies. In addition, pre-planned procedures for study clinicians (e.g. continuity of care plan once a patient has completed treatment) were used to ensure patient safety [42].

**2.2:** ***Flow charts and algorithms*:** Several documents [1, 3, 6, 14, 29, 30, 43–46] recommended incorporating flow charts and algorithms, into research staff’s SRMP educational resources. For example, Haigh and Witham [45] developed a flow chart that provided step-by-step guidelines for managing participants’ distress in research group interviews. This flow chart included response stages (e.g. where the researcher activated an intervention based on the level of severity) followed by review or follow up to identify whether another response is required. Hom et al. [3], also recommended the use of evidence-based suicide risk assessment frameworks and clinical decision trees that clearly described how the research staff could determine risk level and subsequent clinical actions.

**2.3:** ***Electronic prompts/pop-up alerts*:** The use of electronic prompts and pop-up alerts were identified in two documents [30, 46]. The key features of electronic SRMP used by Herbeck Belnap et al. [30] included use of automatic prompts, electronic documentation, use of skip patterns and availability of contact information of on call clinical staff. These prompts and alerts included measures of risk assessment and risk determination. Based on the responses recorded during risk assessment, the SRMP automatically assigned a risk level for self-harm behavior as well as indicated actions to be taken at each level. This strategy is particularly useful for research staff as it streamlines decision-making by providing clear instructions at each step and reducing additional questioning of participants [30].

**2.4:** ***Templates and general principles*:** The use of templates and general principles in SRMPs assist with streamlining research processes and provide consistency and clarity for research staff [1, 16, 33, 47]. Sample plain language templates that include standardized information about study requirements, potential risks, and the right to withdraw from research activities should be available to the research staff for use with participants [16, 33]. General principles for responding to AEs (e.g. the RA is to remain calm, reassure the patient, and contact the supervisor for assistance) also have been included in SRMPs [1].

### Component 3.0: educational resources: Research participants

Similar to educational resources targeting research staff, specific resources for research participants were suggested for SRMPs, including: (3.1) written materials; (3.2) immediate support services; (3.3) available mental health services; and (3.4) a social support person. Of the 36 documents reviewed, 25 (69%) included educational resources for research participants as a core component (see Table 1). Each of these resources are described in more detail below with reference to the key documents describing them.

### Educational resources: Research participants strategies

**3.1:** ***Written materials*:** Written resources, such as information sheets or help pages allow participants to access mental health resources, if necessary [3, 15, 16, 32, 33, 35, 36, 42, 43, 48–51]. Hom et al. [3] indicated that resources and referral information distributed to participants by research staff can provide an opportunity for safety planning and linking with existing social supports to reduce risk. Other examples of written materials included: a suicide referral card [32], an information card with mental health resources [36], and a list of referral information or a referral document [35, 51].

**3.2:** ***Immediate support services*:** Several documents (see Table 1) recommended including information on immediate support services for research participants who may be in acute distress or require urgent mental health interventions [1, 2, 14, 34, 40, 43, 44, 46, 47, 49, 50, 52, 53]. Immediate mental health support services endorsed by research staff included the use of a 24-h help-crisis line [52] and directing those in crisis to a URL link that consists of relevant resources [47]. If such strategies are deemed inadequate for the research team to administer, mobilizing outside resources from a mental health service provider may be a more appropriate option [1, 53].

**3.3:** ***Available mental health services*:** In addition to immediate support services, if a member of the research team identifies a participant is at risk, several documents describe the importance of ensuring research participants are provided with resources to indicate where they can access available mental health services in a variety of settings (i.e. hospitals, community agencies, and educational institutions) [2, 3, 14, 16, 41, 43, 49, 52–54]. More specifically, Bucy et al. [52] described providing information on available mental health resources for research participants; Stanton et al. [49] described providing available mental health resources through individual discussion, written and oral announcements; and others either included debriefing sessions or the option of debriefing sessions with participants [43]. Wilson and Christensen [54] recommended follow up with research participants, who were identified as at risk for suicide and self-harm, 1 week after their research assessment to monitor suicidal ideation and to further reinforce the need to link with available mental health service providers, if necessary.

**3.4:** ***Social support person*:** The use of social support offers research participants the opportunity to have a support person if needed such as family, peers, treatment providers, or local community contacts [2, 3, 42, 52]. As part of safety planning for participants who are at risk for suicide, researchers encouraged participants to identify and provide contact information for an existing social support person [3, 42]. This individual should be reachable during research assessments to assist with the coordination or linking of participants to mental health resources or other social supports in the event of an emergency [3, 42].

### Component 4.0: risk assessment and risk management strategies

It is important for research teams to have the knowledge and skills to identify potential suicide risk level and to act, if required. Strategies include: (4.1) implementation of a structured and explicit SRMP (4.2) identification of risk level; and (4.3) utilization and scoring of validated risk assessment measures. Of the 36 documents reviewed, 27 (75%) included risk assessment and management strategies as a core component (see Table 1). Each of these strategies are described in more detail below with reference to the key documents describing them.

### Risk assessment and risk management strategies

**4.1:** ***Implementation of a structured and explicit suicide and risk management protocol*:** Several documents highlighted that when conducting mental health research where suicide is a potential risk, research teams should have a structured protocol in place to assess suicidal ideation [6, 14–16, 29, 31, 32, 34, 36, 38, 50–52, 55]. According to Lakeman and FitzGerald [16] and Bucy et al. [52], SRMPs are designed to ensure adequate resources or a contingency plan to optimize participant safety. The Committee for Protection of Human Subjects [51] recommends frequent “check-in” notes embedded within the instruments or questionnaires used. These notes capture information including a research subject’s desire to continue to participate in the study or to be connected with a mental health professional that can provide them with referral information. Another example of structured SRMP includes the University of Washington Risk Assessment Protocol (UWRAP) that provides guidelines on determining participant risk level and interventions for staff to offer to participants [14, 15].

**4.2:** ***Identification of risk level*:** Luxton et al. [42] highlight that suicide risk levels are dynamic and change frequently. Thus, as highlighted in 12 documents [3, 30, 34, 36, 37, 39, 42, 44, 47, 50, 51, 54] risk assessment needs to be an ongoing process during research assessments. Three of the documents focused on assessing and allocating established levels of risk to research participants [30, 42]. For example, Luxton et al. [42] defined four levels of risk ranging from low to high with specified clinical response for each level. Furthermore, Wilson and Christensen [54] recommended that assessing suicide risk should include evaluating the frequency and intensity of suicidal ideation, whether the participant has formulated a plan for harming themselves, and their perception of intention to self-harm. Research studies that incorporated telephone interviews, established a set of a pre-identified risk triggers and treatment session checklists to determine participants’ level of risk [42, 50].

**4.3:** ***Utilization and scoring of validated risk assessment measures*:** In total, 11 documents utilized the administration and scoring of a validated risk assessment measure to assess for the presence of mental health conditions (e.g. depression), suicidal ideation or self-harm behaviours associated with increased risk of suicide attempts and death by suicide [14, 30, 32, 33, 35, 37, 43, 46, 50, 52, 55]. The results of these measures provide research staff with another level of information needed to respond. Bucy et al. [52] emphasized that immediate scoring of risk level using a validated measure is essential to ensure the safety of research participants. Specifically, their protocol includes immediate scoring of the Patient Health Questionnaire-9 (PHQ-9) to evaluate the severity of depressive symptoms, the presence of suicidal ideation, and participant needs. Based on the participants’ response, the protocol directs the RA to ask follow up questions and to provide participants with mental health resources. Similarly, clinical research staff responsible for conducting study assessments including reviewing the results of self-report risk assessment measures (e.g. Scale for Suicidal Ideation-Current [SSI-C]) are required to follow a stepped process if the participant is identified as at imminent risk (e.g. self-harm or suicide attempt) [37]. For instance, the study assessor records all potential risks, suicide intent, and protective factors, and then informs the participant’s treatment team at the hospital [37].

### Component 5.0: clinical and Research oversight

Ensuring that monitoring and oversight are established in mental health research was described by 16 /36 (44%) of the documents (see Table 1). Strategies to achieve clinical and research oversight include: (5.1) the use of a data and safety monitoring board (DSMB) or a data monitoring plan; (5.2) safety and risk monitoring of research participants; and (5.3) clinical back-up during research assessments. Each of these strategies are described in more detail below with reference to the key documents describing them.

### Clinical and Research oversight strategies

**5.1:** ***Use of a Data and Safety Monitoring Board (DSMB) or a data monitoring plan*:** Researchers conducting mental health research are often mandated by their institution to use a DSMB [1–3, 6, 29, 31, 56]. The monitoring of study documents and study data is conducted as described within the study protocol to ensure participant safety (e.g. through the identification of adverse events and accuracy and consistency of data collection) [56]. Hom et al. [3] described that DSMBs can offer valuable insight regarding conducting research with participants at risk. Specifically, they recommend that researchers should consult and seek approval regarding their SRMPs from their established DSMBs prior to their use.

**5.2:** ***Safety and risk monitoring*:** In total, six documents highlighted the need for research staff to conduct regular monitoring of participants [6, 16, 29, 37, 46, 56]. This includes conducting follow-up assessments/check-ins throughout the course of the study period to identify potential mental health concerns and ensure participant safety [29]. The National Institute of Mental Health (NIMH) [56] highlights that this responsibility could be assigned to a medical monitor whose role would be described within the detailed study protocol. If there is concern that the research participant’s mental health is deteriorating or they have been identified as high risk for potential suicide, a defined risk assessment with timely and appropriate follow-up procedures should be conducted [56]. More specifically, Ghahramanlou-Holloway et al. [37] describe how they employ safety and risk monitoring during a telephone follow up interview when research staff (e.g. research assistant) become aware of a participant’s imminent risk for suicide. They follow emergency procedures and request assistance, first from the PI followed by the research coordinator. A licensed clinician is also available to deal with any psychiatric emergencies identified by research team members.

**5.3:** ***Clinical back-up during research assessments*:** Several documents [1, 6, 14, 29, 30, 37, 38, 44, 50, 54, 56] note that providing clinical back-up delivers timely support and guidance to research staff during research assessments if a participant is in crisis. Clinical back-up by licensed clinicians (e.g. psychologist, physician, nurse, social worker) provides access to timely clinical assessment and management of high-risk participants [29]. As well, Iltis et al. [6] recommend that PIs, team leads, or senior investigators should be available for decision-making and troubleshooting around emerging safety issues. Crisis counsellors, to support the participant in coping and developing a support plan, are also recommended for research studies, which may recruit study participants with a history of suicidal ideation [50].

## Discussion

This scoping review identified 22 peer-reviewed articles and 14 grey literature documents that aimed to identify core components and strategies included in SRMPs to assess and manage risk and safety for research participants. Acknowledging a need for and creating accepted guidelines for SRMPs to address suicide and risk for mental health studies should not be mistaken with the false notion that there is an inherent risk in conducting mental health research. Beliefs about exaggerated risk are maintained through assumptions that participating in mental health research, particularly suicide-related research, increases the risk of suicidal ideation and behaviour [57–62]. A meta-analysis by Blades et al. [48] demonstrated that asking participants about suicide provided small, yet significant, benefits to participants. Misconceptions on the degree of risk in mental health research has negative implications on research proposal submissions to REBs, where participant safety is the primary concern.

There are many collaborative efforts to standardize research processes around the world. However, specific components and strategies to include in SRMPs to mitigate suicide and risk in mental health research are lacking. This review revealed that SRMPs provide vital information for research teams on how to assess and manage risk and respond to research participants in crisis. Our review identified five core components that researchers, research agencies, and REBs commonly identified as essential in the development and administration of SRMPs. These components included: (1) training; (2) educational resources for research staff; (3) educational resources for research participants; (4) risk assessment and management; and (5) clinical and research oversight. All five core components should be considered when conducting mental health research to ensure the safety of the research participant and rigor of the research. An array of strategies related to each component were identified that could be selected or tailored across a variety of research contexts. However, there are few documents that collectively recommended implementing all five core components and relevant strategies [3, 16]. This is surprising given the importance of ensuring participant safety and highlights the need for REBs within organizations to standardize SRPMs across research projects. More research is required to verify the core components and strategies, including how to tailor them across different stakeholder groups and contexts.

Training was identified as an essential component of SRMPs for all research teams conducting mental health research on participants at risk for suicide [3]. Training is generally seen to have a vital part in achieving organizational goals in healthcare [63] and is a catalyst to staff efficiency, career development, and job satisfaction [64]. In mental health research, training needs to include specific direction on risk assessment and how to handle a variety of different situations such as participant’s report of suicide ideation and/or action [29]. Research team leads (e.g. PI, research associate) need to ensure all research staff are trained in how to respond to suicide and assess risk. Campbell et al. [29] stress that training activities need to be extensive and varied. They strongly recommend that sufficient funds are allocated for development of suicide and risk management learning resources.

While some training strategies were frequently cited in the reviewed documents (e.g. follow-up and debriefing with research staff [2, 28, 30]), others were only mentioned in a single document (e.g. team building [29]). This lack of consistency in identifying specific training strategies could suggest that SRMPs are at different stages of development based on the type of study being conducted, source of funding, and organizational training requirements. SRMPs may also be utilized differently depending on the expertise and availability of the research staff. Furthermore, while some training strategies were described in depth (e.g. three-day workshop [29]), others lacked clarity and precise details (e.g. establishing relevant experience, delineating roles, and ensuring qualifications for the research team [38]). Inconsistencies in training details provided throughout the reviewed documents highlights the need to determine the aspects of training that are of greatest benefit across diverse research settings in order to develop standardized training modules for assessing and responding to suicide risk that can be implemented similarly to training in other research procedures.

Strategies addressing the essential qualifications for research team members to assess risk of suicide, was primarily targeted towards the PI or team lead (e.g. research associate). Specific research and/or clinical qualifications (e.g. professional licensure) and experience for team member roles (e.g. research assistant, research coordinator, trainees) was either absent or vague [38]. The sufficiency of required knowledge for various team members was also not specified. Better descriptions of qualifications and knowledge requirements for all team member roles would assist with tailoring the research training for individual projects. Ongoing leadership training also needs to occur to ensure that an individual with the required qualifications and knowledge is appropriately designated to identify training needs, provide training for the research team members and build in time for follow-up of the research activities. Davidson et al. [65] suggest that successful leaders in health research organizations need to be skilled and competent to enhance collaboration, communication, team effectiveness, data quality, and scientific productivity.

Educational resources are another key component recommended and used in the reviewed documents, to contribute to a safe environment for both individuals (e.g. research staff and research participants) and organizations (e.g. REBs) [14]. The use of evidence-based protocols and educational resources is consistent with effective knowledge translation strategies that support the successful implementation of research evidence in practice [66]. Hom et al. [3] draw attention to the fact that even though individuals and organizations may be wary about research involving participants at risk for suicide, evidence-based protocols to assess and manage this risk can be effectively adapted for different research contexts. The evidence supporting educational strategies and resources needs to be updated regularly by a responsible person to conduct the updates. This individual will need a degree of information literacy to decipher and interpret the immense amount of knowledge that is generated through healthcare research [67].

Educational resources have been developed for research staff to support their knowledge of, and use of, study procedures that outline to manage and respond to participants in crisis [1, 29]. Strategies such as flow charts and algorithms (Strategy 2.2) promote a standardized approach to managing the research processes and aid in decision making. Vannoy et al. [1] state that although flow charts for managing suicide risk and management are rare, they are particularly useful in outlining decision-making around “specific actions to be taken, when, and by whom” (p. 5). Automatic assignment of risk mentioned in Strategy 2.3 [30] also supports standardized decision making. However, this strategy could be prone to malfunction (and require technical intervention) and may not consider all pertinent information related to clinical context and judgment.

Educational resources for research participants are also important to ensure they are well informed about the study and its potential risks, and that they have knowledge about how to access immediate support and available mental health services, if required. Written educational strategies (Strategy 3.1) have similar limitations for participants as for research staff. They need to be developed, specifically targeting the participant stakeholder group, written in plain language, and address key messages of importance for this group [16, 68]. Strategies that address the need for immediate or available mental health services [43, 52] should be well researched, current, and accurate. Providing this information to participants may be considered a co-intervention that may impact study results. Achieving a balance between providing mental health information to keep the participant’s safe and maintaining the study rigor may be challenging. The content of the information given to research participants is important for researchers to track and disclose. Identification of a social support person is also appealing, however studies do not clearly provide information on social support or “support person” or how participants may benefit from reaching out for this support [3, 42]. Operationalization of social support as part of SRMPs is also not well described in the reviewed documents and the logistics for implementation are lacking.

This review highlights the need for research staff to be trained on how to identify suicide risk level and to respond accordingly. Strategies that could assist in achieving this would include developing a structured and explicit SRMP (Strategy 4.1) within the study proposal and utilization of validated risk assessment measures (Strategy 4.2). These strategies, however, depend on having knowledgeable and capable researchers and clinicians as part of the research team to develop SRMPs and to conduct training with research staff. This training should take into consideration different levels of risk and diverse levels of research staff knowledge and qualifications [69]. Across documents, levels of risk were mostly matched with required interventions [30, 42]. Strategies tailored to risk severity can be advantageous to ensure participants receive adequate treatment while also allowing research staff to make decisions with confidence.

Among the components and strategies identified in this review, there was a lack of input from research participants and/or stakeholders described in the development of SRMPs. This is an important consideration for future SRMPs in order to be participant-oriented and align with their needs. Manafo et al. [70] highlighted significant momentum for engaging patients in all phases of the research process. Working collaborations between patients and researchers has been shown to improve patient and health care outcomes [71]. With increased focus on patient engagement in clinical research [70], it is imperative to include participant perspectives in the development of research documents such as SRMPs. Enhancing the value of SRMPs will largely depend on careful evaluation by multiple stakeholders.

While this review found two articles reporting best practices for online SRMPS [30, 47], there remains a lack of literature on how to adequately prepare researchers and REBs for conducting mental health research involving e-health applications [72, 73]. As mental health research is rapidly evolving, the utilization of technology and online clinical research studies has become more common as care delivery approaches [74]. Specifically, research involving e-health interventions, while promising as sustainable and scalable [74], raises questions on how to ensure participant safety when in-person and face-to-face interactions do not occur [72]. With online administration of e-health applications in mental health research, there is a need to have appropriate safeguards in place to clearly lay out the best course of action during unanticipated and crisis situations. Defining specific criteria required in SRMPs used in e-health research studies, including online administered and digital interventions, could eliminate uncertainties and relieve the burden currently placed on both REBs and mental health researchers.

### Limitations

While this review has several strengths, including the use of an iterative search strategy and a range of databases, there are limitations to note. First, the use of varying terminology to describe SRMPs in the literature (e.g. SOP, action plan, safety protocol, high risk management procedures, suicide risk assessment) may have contributed to the limited number of documents identified for this review. Inconsistent terminology can also impede the development of standardized SRMPs where there needs to be clarity on processes such as risk assessment and determination of adverse effects. Second, the language of included documents was limited to English; therefore, relevant non-English documents may have been missed. Third, the study participants of included documents was limited to age 16 years and up. Important considerations from younger populations (e.g. under 16 years of age) may have contributed more information about potential strategies to support the implementation of SRPMs for children and adolescents at risk.

## Conclusions

This scoping review, including peer reviewed academic literature and grey literature documents, identified five core components to include in the development of SRMPs for mental health researchers. Multiple documents described a range of strategies to address individual core components. There are few documents that collectively implement all five core components and relevant strategies. Ideally, these findings could inform the development of SRMBs for research teams and REBs.

## Supplementary Information

**Additional file 1.**

## Data Availability

There is no study data as this is a scoping review but the search strategy is available as an Additional File.

## Abbreviations

PRISMA-ScR: Preferred Reporting Items for Systematic Review and Meta-Analyses extension for Scoping Reviews
PI: Principal investigator
REB: Research ethics board
SOP: Standard operating procedure
SRMP: Suicide and risk management protocol
