# Supplementary material for: Core components and strategies for suicide and risk management protocols in mental health research: a scoping review
Source: BMC Psychiatry. 2021 Jan 7;21:13. doi: 10.1186/s12888-020-03005-0 (PMC7792084; doi:10.1186/s12888-020-03005-0)
Supplement: Supplementary file 1 — Additional file 1. [file 12888_2020_3005_MOESM1_ESM.docx]

**Additional File 1**

**Search Strategy for Medline-OVID**

Database: Ovid MEDLINE(R) <1946 to January Week 1 2020>

Search Strategy:

--------------------------------------------------------------------------------

1 standard operating procedure*.mp.

2 standard work procedure*.mp.

3 safety plan*.mp.

4 suicide risk management.mp.

5 risk management/ or risk assessment/ or risk factors/

6 professional ethics/

7 ((suicid* or safety or risk or crisis) adj3 (procedure* or protocol* or intervention* or guideline* or

parameter* or management or response*)).mp.

8 or/1-6 [Safety protocols]

9 exp Research Subjects/

10 ethics committees, research/

11 human subject*.mp.

12 experimental subjects/ or behavioral research/

13 (research* adj2 (setting*or staff or participant*)).mp.

14 or/9-13 [research subjects]

15 suicide/ or suicidal ideation/ or suicide prevention/

16 mental health crisis.mp.

17 self-injurious behavior/

18 self-harm.mp.

19 (suicid* adj2 (risk* or sign* or ideation* or attempt*)).mp.

20 or/15-19 [risk of harm]

21 8 and 14 and 20

***************************
